# First Step to empowering change: enhancing self-efficacy, energy management, and physical activity in patients with sleep apnea

**DOI:** 10.3389/fresc.2024.1359371

**Published:** 2024-07-12

**Authors:** Gitte Johannesen, Anders Ravnholt Schüsler Damlund, Sofie Grundtvig Vinter, Helle Skadborg Spuur, Mathias Sarkez-Knudsen, Thora Grothe Thomsen

**Affiliations:** ^1^ Department of Occupational and Physiotherapy, Zealand University Hospital, Køge, Denmark; ^2^ Department of Occupational and Physiotherapy, Zealand University Hospital, Roskilde, Denmark; ^3^ Department of Otorhinolaryngology, Zealand University Hospital, Køge, Denmark; ^4^ Department of Regional Health Research, University of Southern Denmark, Odense, Denmark

**Keywords:** rehabilitation, activities of daily living, sleep apnea, patient-centered intervention, self-efficacy, energy management

## Abstract

**Introduction:**

Fatigue often leads to a sedentary lifestyle, negatively impacting health, mortality, and quality of life. Engaging in physical activity can be challenging for individuals experiencing fatigue, particularly those with sleep apnea. This study's objective was to assess the “First Step” concepts’ usability in constructing and implementing different interventions through qualitative data. The intervention targets patients with sleep apnea, focusing on individualized energy distribution and meaningful engagement in physical activity.

**Methods:**

Two programs were developed based on the First Step concept, a rehabilitation program and a patient education program. Initially, 13 patients were recruited, split between the groups, with two dropping out. Primarily evaluated through qualitative data, patients in both groups attended group interviews. For one of the programs supplementary quantitative data were collected through the 6-min walk test (6MWT), Sit-to-Stand test, and Canadian Occupational Performance Measure (COPM). Patients in the rehabilitation program also monitored daily step counts using activity trackers.

**Results:**

Patients found the energy management education enlightening, enabling them to make conscious changes in their daily lives. They reported the program's positive reception, with social interaction playing a crucial role in its success. Of the six patients who completed the rehabilitation program, significant improvements in 6MWT scores were observed, indicating enhanced walking endurance. While no changes were seen in the Sit-to-Stand test, COPM results showed notable improvements in performance and satisfaction with chosen activities.

**Discussion:**

The incorporation of the First Step concept empowered patients with sleep apnea to manage fatigue, conserve energy, engage in meaningful activities, and improve their wellbeing. Merging occupational therapy and physiotherapy interventions effectively addressed daily challenges while promoting physical activity. Adaptations to the program, guided by patient feedback, suggest a preference for longer, more personalized sessions. This approach offers a promising pathway to improving quality of life for individuals with chronic conditions.

**Conclusion:**

Our study highlights the usability of the First Step concept, integrating occupational therapy and physiotherapy, to address challenges in individuals with sleep apnea. The tailored, multidisciplinary intervention prioritizes meaningful activities, focuses on energy distribution and physical exercise, yielding improved satisfaction and performance. Further research is warranted to enhance this salutogenic approach for chronic conditions.

## Introduction

People with more than one chronic disease, that is, multimorbidity, are prone to lead lives with less physical activity than others (1). The sedentary way of life has an impact on their general health status, mortality, and quality of life (2). Nevertheless, it is often a great challenge for this group of patients to engage in rehabilitation and increased physical activity, given they are already challenged by everyday life activities (2). As part of a larger European Union (EU)-funded Interreg Öresund-Kattegat-Skagerrak (ÖKS) project, Sleep Across Waters (3), this study sought to verify the relevance of a new concept, called “First step.” The concept combines a focus on the individuals’ distribution of energy with the engagement in physical activity and focusses on interventions that are perceived as meaningful and empowering to the individual. The intervention targeted a group of patients, in which many have more than one chronic disease, patients with obstructive sleep apnea (OSA).

Patients with obstructive sleep apnea are commonly over the age of 50, the majority of them are obese and they may have comorbidities such as pulmonary or cardiovascular diseases (4). Living with sleep apnea often results in poor quality of sleep and fatigue during waking hours (4). This daily fatigue can lead to a decreased quality of life (5). OSA can lead to cognitive dysfunction, affecting, among others, memory and concentration (6, 7), a decline that can affect a person's ability to manage activities of daily living (ADL) (8). Cognitive dysfunction in patients with OSA can further impair their ability to engage in physical activity and participate in ADL (8). This can create a cycle where reduced physical activity worsens OSA symptoms, including cognitive dysfunction, further reducing the motivation and ability to be active. A meta-analysis found that an exercise intervention could contribute to an increase in quality of life, a decrease in apnea-hypopnea index score, and a reduction in daytime sleepiness greater than what is found from use of continuous positive airway pressure (CPAP) (5). O’Donoghue and McKay found that patients reported sleep apnea as a life-changing condition that affected daily life and occupational engagement, emphasizing the need to empower patients to manage their condition (9).

The NICE guidelines recommend life style changes as an important part of sleep apnea treatment, such as weight loss, increased physical activity, and smoke cessation (10). This goal of ensuring lasting life style changes as well as engagement in a comprehensive treatment program may be difficult to reach without the intervention being rooted in patients’ daily lives. To help the individual embrace physical activity, target the life-changing factors and promote management of ADL, this study developed an intervention, based on the new concept First Step. The concept is based on a combined effort by occupational therapists (OT) and physiotherapists (PT) and has been developed at the Department of Occupational and Physiotherapy at Zealand University Hospital in Denmark.

The study investigates the potential of applying the First Step concept to an intervention targeting patients with sleep apnea, a group known to suffer from fatigue. The First Step concept combines a focus on the individual's energy distribution with interventions that are perceived as meaningful and empowering. The Sense of Coherence (SOC) theory informs the patient approach, aiming to motivate patients in a way that aims to be comprehensible, manageable, and meaningful. First Step also draws on principles of occupational therapy (activity analysis and energy distribution) and physiotherapy (optimizing physical function) to address physical limitations. The primary objective is to assess the concepts’ usability, in the construction and implementation of different interventions, through qualitative data.

## Background

### First Step

Based on a salutogenic approach to healthcare, this concept strives to ensure that patient interventions are perceived as meaningful to the individual and based on the individual resources available in the situation. The aim of the concept is to assist the patient in taking the first step toward a better quality of life. This includes a conscious focus on energy distribution before incorporating additional physical activity in rehabilitation and everyday life. It is our profound opinion that to be successful we have to take into account the patient's current physical, mental, cognitive, and social status, as well as their exercises, daily activities, and the settings these are performed in (11). Personal values, habits in everyday life, and motivation for performing physical activity are also of great importance in successful goal-setting, promotion of self-efficacy, and in the intervention as a whole (12).

In Denmark, there's a growing emphasis on improving the quality of life for individuals with chronic conditions. One area of interest is ADL and energy management. ADL has a strong historical connection with occupational therapy, leading many OT professionals to adeptly use activity analysis for energy management in various contexts. A notable development was the release of a comprehensive textbook in Denmark in December 2022 (13). This book comprises chapters contributed by OT experts, elucidating interventions tailored to patients with various challenges. One of the authors of this study is not only an editor but also a contributor to the textbook. The insights derived from employing energy management interventions across varied contexts and to various patient groups significantly informed the conceptualization of the First Step approach.

### Ensuring meaningfulness

The concept of First Step focusses on the idea that to integrate new actions into ones daily life, the actions have to be perceived as meaningful by the individual. In this, the concept is inspired by Antonovsky's theory of salutogenesis (14). A strong focus of the theory is upon meaningfulness, as well as on what resources are available to help the individual fight and cope with sickness and other stressors, with less focus on the sickness and symptoms themselves.

Antonovsky also introduces a continuum, on which every individual moves between “ease” and “dis-ease”, never being only just healthy or sick (14). The salutogenetic orientation makes us concentrate on the factors that strive to move the individual in the direction of “ease” on the continuum, which is also a driver for this study.

At the center of salutogenesis is “the SOC,” which includes the three components of comprehensibility, manageability, and meaningfulness. Meaningfulness and seeing the greater purpose, e.g., in activities of daily life, are described as fundamental factors in motivation; to have areas in life of significance worth investing time and energy on. It is not necessarily the performance of a specific activity in itself that is required to be meaningful to the individual, it can also be what it achieves or represents, e.g., work as a means to support one’s family. SOC is an important determinant in the preservation of the individual's position on the continuum and in the effort to move toward “ease.”

When using the First Step concept on this intervention, the aim is to support the individual’s ability to comprehend, manage, and find better meaning in their life situation, with sleep apnea. This is sought done by the patients identifying what they find meaningful in their daily lives, attaining knowledge about energy distribution, and how it is possible to tailor meaningful physical activity to meet individual needs.

### Energy distribution, motivation, and goal-setting

If patients are to be motivated to change their daily lives with meaningful graduations, additions, and reductions of activities, the goal of the change has to be the driving factor. In the First Step concept, the focus on energy distribution, motivation, and goal-setting in the intervention is based on occupational therapy theory and tools developed to support this approach.

The Model Of Human Occupation (MOHO) (12) is an occupation-based theoretical model that offers an understanding of how people are motivated to undertake activities by factors related to personal characteristics. By analyzing, e.g., how a person finds meaning in the activities they choose to do, their values, interests, and performance skills, the OT can identify potential opportunities to improve the performance by graduating or adapting the activity and thereby attaining better energy distribution (15). The aim is to help the person experience success in performing the activity, motivating the individual to repeat the experience, and build future habits upon good experiences with customized (physical) activity.

**Figure 1:**
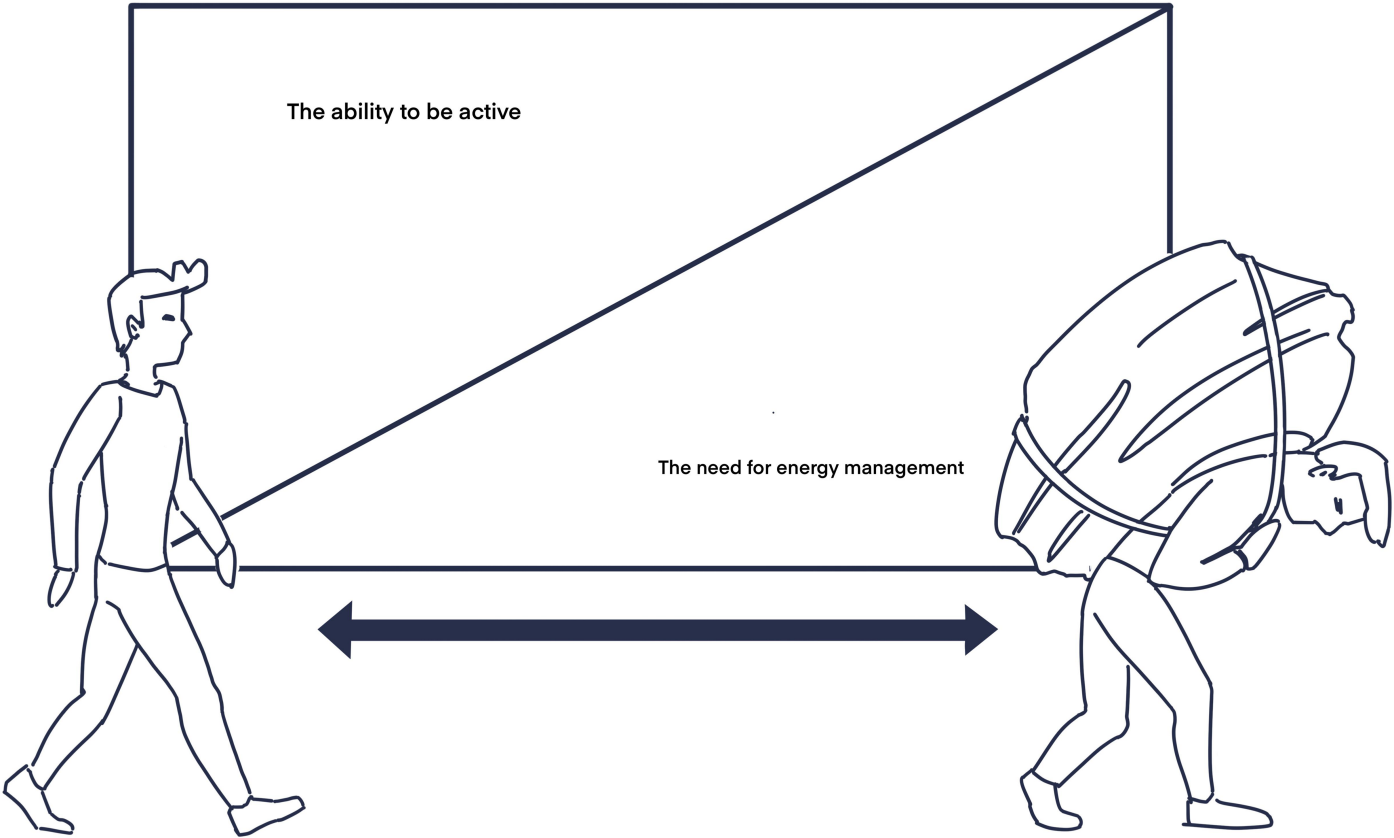
The AEM model—the relation between the ability to engage in physical activity and the need to focus on energy management in everyday life.

Self-efficacy is a key concept in MOHO (9) as well as in First Step. It includes the individuals’ sense of self-control and to what extend he or she has the capability to perform the activities they wish to. The loss of function due to chronic disease has been shown to be linked to the individuals’ sense of self-efficacy (16). To work with establishing new habits and activity patterns it is essential to focus on strengthening the persons’ self-efficacy. This will increase the possibility of them prioritizing the activities they find meaningful and to stay motivated to continue carrying them out in the future. Individual goal-setting that targets these exact activities plays an important part in the process (17). Learning how to actively focus on energy distribution will also support the individuals’ self-efficacy as it creates recognition and awareness and offers the tools needed to manage everyday life. The need for focusing on energy distribution will increase as the total burden of the physical, mental, and social conditions affects the individuals’ ability to act, as is illustrated in Figure 1.

### Developing an intervention

It is well known that physical activity is the key to good health (18). However, as illustrated by the Active Energy Management (AEM) model in Figure 1, the load of various conditions such as sleep apnea can decrease the ability to participate in physical activity. This may be due to fatigue, lack of motivation, and general lack of energy (19). The decreased ability to engage in physical activity increases the need for focusing on energy management.

This study set out to test the First Step concept on a relevant group of patients, where the goal was to give patients the tools and opportunities to manage their energy better in everyday life to make room for increased physical activity. This was done with a specific focus of grading and adapting ADL (20, 21) and the use of specific energy conservation tools, most of which involve goal-directed prioritizing and planning.

**Figure 2:**
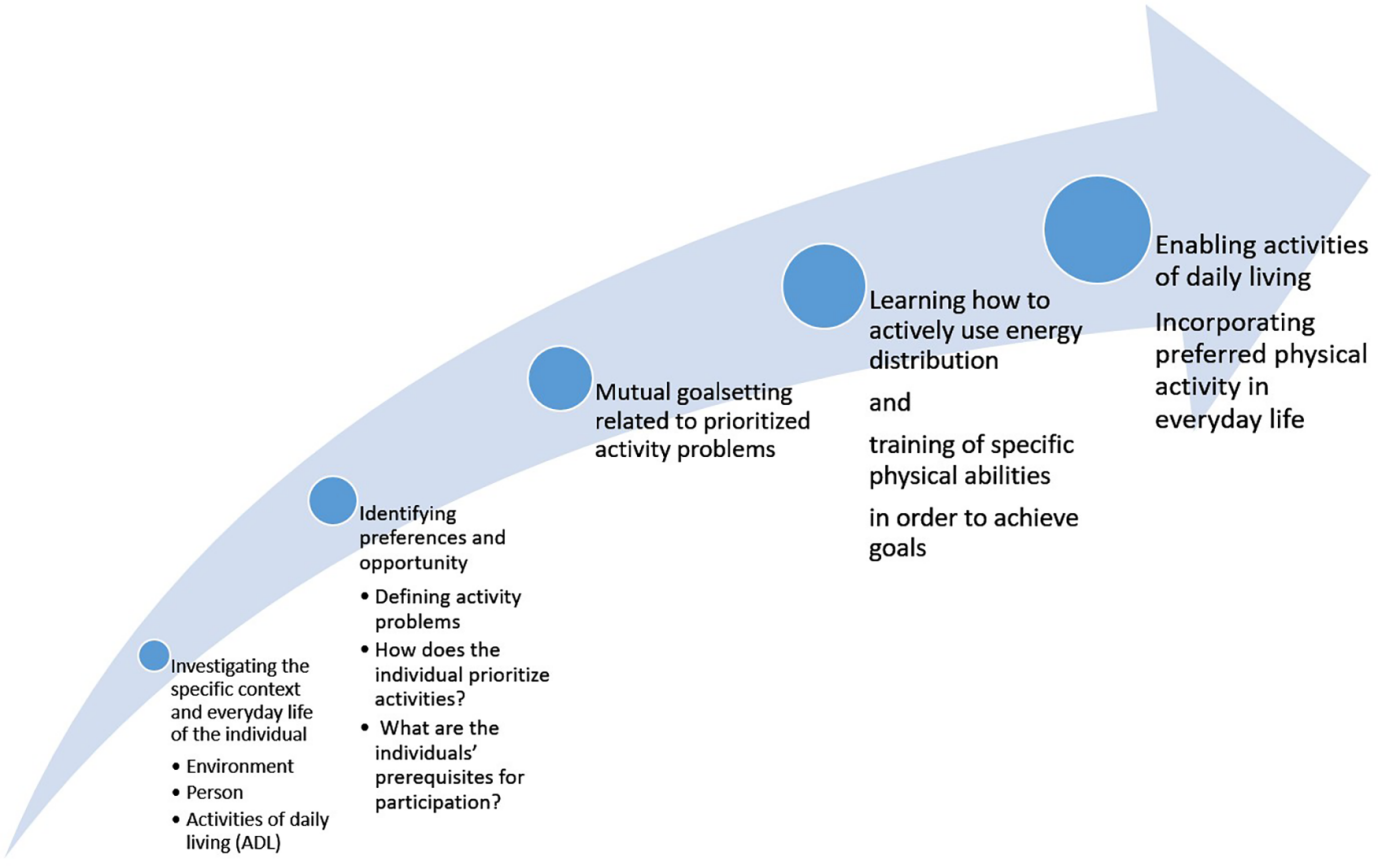
Focus in different phases of the First Step–inspired intervention for patients with sleep apnea.

The intervention was inspired by a combination of models of occupational therapy practice and physiotherapy principles of physical activity, as it required a multidisciplinary approach to treating and empowering patients. Inspired by the First Step concept, the intervention in the rehabilitation program was planned to start by investigating different elements of the individual person, activities of daily life, and the context in which they spend their lives (Figure 2). Hereafter each patient would be facilitated in identifying and defining activity problems that they found to be of highest priority to improve. Mutual goal-setting was to be completed at the outset, after identifying the activity problems. Subsequently, the patients were to learn about the use of energy distribution and how exercise can support betterment of activity problems, as well as be integrated into the schedule of everyday energy expenditure without this causing further exhaustion. By using these acquired skills, the patients should be able to achieve the goals set for the course and perform ADL better as well as be able to incorporate physical activity into their everyday lives.

## Methods

### Design

Patients participated in one of two possible interventions and subsequently evaluated it through a group interview. The two interventions were based on the most commonly used in the Danish healthcare system after treatment at the hospital; a rehabilitation program and an education program.

### Patients

Recruitment was undertaken by healthcare personnel of the Department of Otorhinolaryngology and Maxillofacial Surgery, who screened patients with moderate to severe obstructive sleep apnea in the course of treatment at the outpatient clinic and contacted the eligible patients. The only requirements for the patients to be eligible were that they had to have been diagnosed with moderate to severe sleep apnea, be ambulatory without walking aids, and be able to speak and understand Danish.

### Interventions

The interventions took place at Zealand University hospital at the Department of Occupational and Physiotherapy. All the sessions were class-based, an OT and a PT were responsible for the classes, and both professions were always represented in each session.

This study explored two different variations on the First Step–inspired intervention program. Both interventions focused on merging daily activities and energy management with physical activity and exercise, with the aim of establishing actions that the patients would be able to integrate into their lives.

### Rehabilitation and patient education program

The programs were designed by using the First Step concept as a framework while merging existing experience with rehabilitation programs and patient education, e.g., concerning patients with chronic obstructive pulmonary disease (COPD), with feedback from the patients.

The rehabilitation program was planned to be a 12-week intervention with two weekly sessions of 1 h each. The patient education was for 4 weeks, with one weekly session of 2 h. The interventions were planned to include the following:

- A Canadian Occupational Performance Measure (COPM) interview pre and post intervention to establish meaningful goal-setting (only for the rehabilitation program).
- Pre and post testing of 6-min walk test (6MWT) and Sit-to-Stand test (only for the rehabilitation program).
- Patient education in the principles and the practical use of energy management.
- Patient education in the treatment of sleep apnea, pathology, and guidance in use of CPAP from a team of multidisciplinary health professionals (doctors and nurses).
- Physical exercise during the weekly session as well as home-based exercises chosen to support the initial goal-setting for the individual.
- Homework related to energy management.

Inspired by Jurado-García et al., who successfully used a graduated walking program to decrease apnea-hypopnea index over 25 weeks (4), the rehabilitation program also had a focus on daily step count and in this way aimed to make small but significant increases in everyday physical activity. The patients who took part in the multidisciplinary rehabilitation program were issued an activity tracker (Fitbit Charge 5) to monitor their daily step count. The daily step count was self-reported in a patient journal. Patients were instructed to go on walks every day and do home exercises when they were not attending sessions at the hospital. If patients were unable to attend class, either an OT or a PT contacted them on a weekly basis.

Data collected from COPM, 6MWT, and the Sit-to-Stand tests were to be used only supplementary to the study's main findings from the group interviews. With a small study population quantitative data could contribute with insights into the potential positive effects of the intervention, which could be explored further in future studies.

COPM is an interview-based tool that is used to identify highly prioritized, individual everyday activity problems that limit a patient's participation in daily life. At the assessment the patient rates the activities according to importance on a 10-point rating scale; the patient is asked to choose up to five of the most important problems. The first assessment concludes with the patient rating their own performance and their satisfaction with the performance for each of the five problems. In this intervention the chosen activities were used to set individual goals for the course, making the activities and exercises relevant and meaningful to the patient.

### Introducing tools to manage energy distribution

Energy conservation techniques are widely used to optimize daily life for people with many different diseases and conditions (22, 23). Seven principles of energy conservation, used in lung rehabilitation (24), were presented to the patients by the OT. These are prioritizing, planning, pacing, breathing techniques, positioning, rearranging surroundings, and the use of assistive devices. Specific tools related to prioritizing and to planning were used. The tools are known from OT interventions related to different groups of patients suffering from fatigue (24). The “Priority diagram” helps the patient identify and recognize the balance between the activities that add to their energy and the activities that drain their energy. The “Energy balances schedule” illustrates the patient's perception of their use of energy on activities during the day and the week. The purpose of using these tools is to help the patients acknowledge what ADL they find meaningful, their values, interests, and habits as well as making it possible to target and help manage energy expenditure. The patients were encouraged to fill in the diagram and schedule at home, as preparation for the forthcoming sessions and ongoing follow-up to the subject during the course.

### Adapting the intervention

With the First Step approach, great care was taken to ensure that the patient saw the intervention as meaningful, manageable, and motivating. This is reflected in the many adaptations that were made to the intervention. Some are presented here.

To accommodate that some patients might experience daytime sleepiness (8), patients were asked what time of day the group sessions would suit them best. The feedback highlighted that two sessions a week was too much to cope with and that the scheduled time for attendance had to be no sooner than noontime. To accommodate this the intervention was redirected to two sessions a week for the first 6 weeks and one session a week the following 6 weeks; attendance at 1 pm.

After the initial examination of the patients, using COPM, it was clear that many had issues with activities of daily living. These activities were also rated as very meaningful and important to them personally. Examples were problems with housekeeping, activities that required reaching the floor, and carrying a load. Younger patients (40–50 years old) expressed difficulty with self-care and getting dressed. These findings supported the need for the OT to focus even more on activity analyzing and grading to provide the tools to manage everyday life in a less energy-consuming way.

In the course of the program there were other actions undertaken to meet the individual needs of the patients. In regard to physical exercise, the content was customized to fit the needs and goals of the patients. An example of this was a strong focus on the ability to get down to the floor and get up again, as this was a problem for several patients. Another example was that two patients were instructed on how to walk their dog in shifting intervals of high and low intensity, as it was difficult for these patients to incorporate the daily physical exercise in any other way. One patient expressed difficulty incorporating and making room for the desired physical activity in his everyday life. This inspired the making of individual weekly schedules for every patient, planning the week's activities in a way that made room for both exercise and physical activities of higher intensity as well as resting.

### Evaluations of the interventions

The evaluations of the interventions were based primarily on qualitative findings. Patient evaluations of the two interventions were conducted through two group interviews following the same interview guide. One group consisted of five patients from the rehabilitation program and the other group consisted of four patients from the patient education program. All the patients were invited to attend the interviews, but only nine were able to participate.

An interview guide was made, based on previous literature and in collaboration with the OT and PT.

The interviews were conducted by two of the authors, GJ and AD. During the interviews, inquiries were made toward patients’ previous experiences with rehabilitation, experience with the current setting, and the content of the interventions. The interviews followed the interview guide, while keeping conversation open, allowing the possibility that the conversation could diverge and follow relevant topics as they arose.

The interviews took place at the hospital after each patient’s last session and were conducted in a neutral location away from the intervention and familiar personnel.

The potential quantitative effects of the interventions were measured using standardized versions of 6MWT (25, 26), 30-s Sit-to-Stand test (27), and the COPM (28–31). Data were collected by SG and HS, who also supervised each group session. All collected data had personal information and identifiers redacted and were stored on a secure server till the end of the study.

## Data analysis

### Results

**Figure 3:**
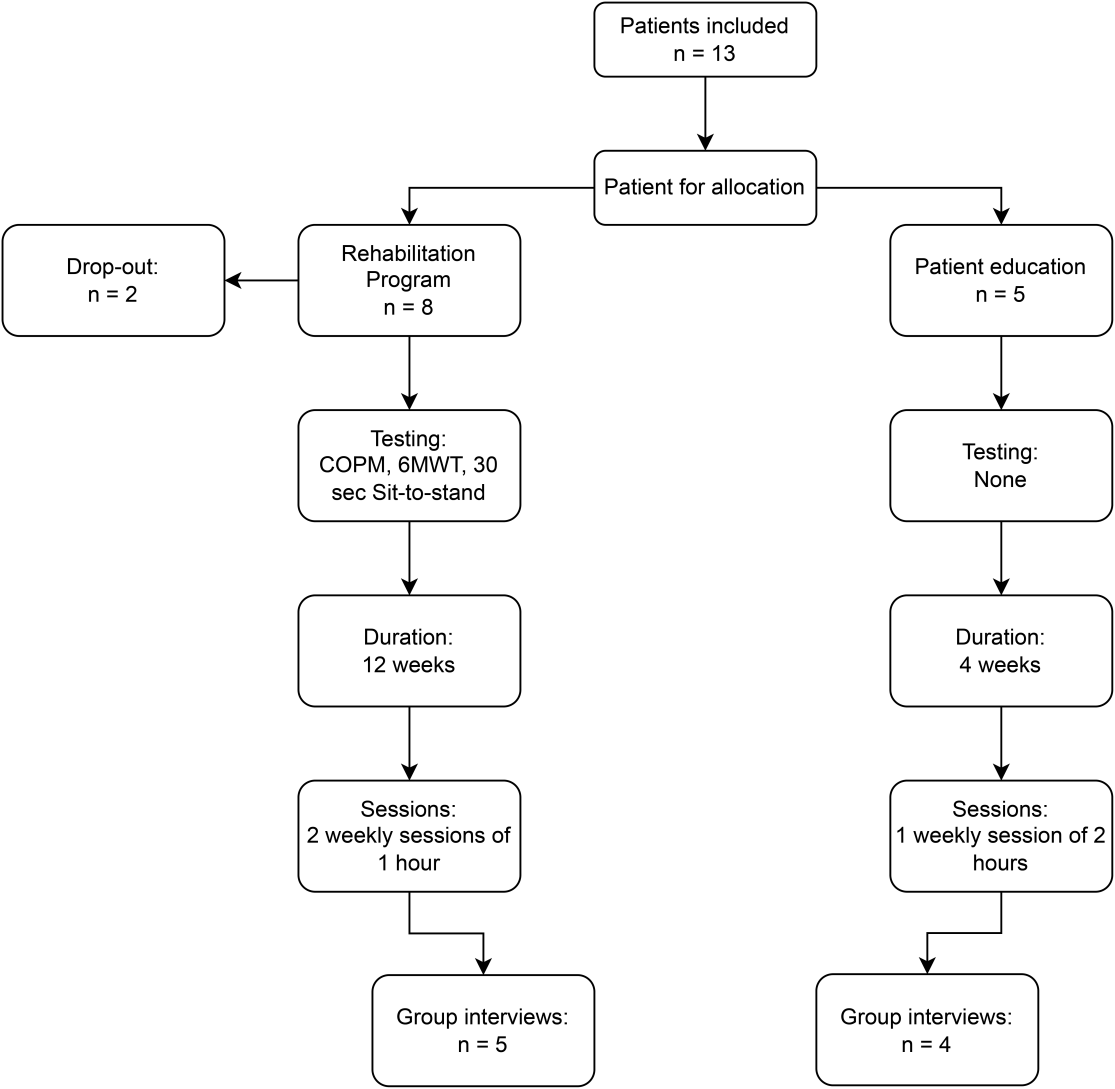
Flowchart of interventions.

Altogether 13 patients were initially recruited to the two programs. Two patients dropped out (15%), one owing to mental health issues (7%) and one because he did not feel the effects of his sleep apnea in his daily living (7%). The remaining patients consisted of six women and five men, with a median age of 67 (38–80) years. The six patients attending the rehabilitation program participated in 6MWT, Sit-to-Sand, and COPM interview pre and post intervention (Figure 3).

### Qualitative data

#### Interview

Group interviews were transcribed *ad verbatim* and analyzed using systematic text condensation based on Malterud (32). The text condensation was driven by an inductive and interpretative approach (33). During condensation emerging themes were gathered and discussed among the primary investigators to get a wider analytical space. The discussion lead to the development of four major themes, encompassing all the emerging themes: Program, Sessions, Energy management, and Sleep apnea management.

#### Program

All patients were pleased with the program and for some the class-based exercise had been the jumping-off point to continue previously discontinued daily exercise programs. Some of the patients also experienced weight loss during the program.

“*It has been built around the individual and the individual's special needs, which I found to be very positive. … They build something structured for us, so that we can continue it after we get home*.” (Male 40–50 years).

In regard to what could supplement the intervention, a common request from the patients was a higher degree of multidisciplinary content from nurses and nutritionists, based on their lingering question on treatment, test results, and diet.

The initial consultation with the OT, including goal-setting, was mentioned as a positive element that contributed to a more focused program for the patient.

Several patients pointed to transportation issues and the time of day as a barrier for attendance and a potential reason to decline the program. Patients with workplace obligations had to rely on a flexible workplace to attend. To decrease transportation and the effect on their work, patients from the rehabilitation program stated they preferred attendance at the hospital once per week and a supplementary session from home.

#### Sessions

To accommodate the need for individualization of the intervention, the patients in the rehabilitation program preferred that sessions went from 1 to 1.5 h. It was a common experience that some elements of the intervention felt lacking when they only had an hour. The patient education had sessions of 2 h each, but the patients expressed that they would prefer shorter sessions if they could receive more sessions over a longer period of time.

Several patients said that even though the patients were of different age groups, they had had a positive experience attending the class. They were able to gain support from each other, by sharing experiences and humor. There rose a consensus that the class was a safe space were they were able to talk about subjects they would not have shared with their family and friends. This also led to a feeling of camaraderie among patients and a request for a support group for patients suffering from sleep apnea, possibly via social media.

#### Energy management

The patients were happy with the energy management education as a part of their rehabilitation. The education and associated homework was an eye-opener for several. A patient reported that she sometimes got angry and frustrated after the sessions, because they stirred something inside her, which she subsequently was grateful for.

“*The program could have been longer, because it really has been an eye-opening experience for me. Some days I have been so angry when I left here, because things had gotten through to me and moved something inside me. Afterward I have been so thankful for that*.” (Female 50–60 years).

Several patients used the supplementary materials in relation to energy management to visualize their daily energy expenditure, making themselves aware of their own prioritization in choosing activities. This gave them the opportunity to make conscious changes for the better.

#### Sleep apnea management

Multiple patients had questions regarding the usage of CPAP and one patient had stopped using it altogether because of the frustrations. There was a desire for additional sparring and the opportunity to ask questions about the equipment. The -patients attending the patient education program had a short session where they were able to ask a nurse and a doctor some questions about sleep apnea and its treatment. They expressed that this had been a positive experience, but that they wanted more time to elaborate on factual knowledge of the condition and ask additional questions.

### Quantitative data

Six patients completed pre- and post-intervention Sit-to-Stand tests and 6MWTs (Table 1). There were no changes in sit-to-stand scores after the intervention (Table 2). Four out of six patients showed improvements in their 6MWT exceeding 50 m (25). This suggests that although not statistically relevant, the quantitative data provides insights into possible benefits from such interventions.

**Table 1:** Test results of 6-min walk test.

6MWT	Pre	Post	Change
P1—Male, 40–50 years	438	480	42
P2—Male, 40–50 years	527	599	72
P3—Male, 70–80 years	191	318	127
P4—Female, 60–70 years	254	442	188
P5—Female, 50–60 ears	438	504	66
P6—Male, 50–60 years	574	611	37

**Table 2:** Test results of 30 Sit-to-Stand tests.

Sit-to-stand	Pre	Post	Change
P1—Male, 40–50 years	7	12^a^	—
P2—Male, 40–50 years	11	15	4
P3—Male, 70–80 years	15	15	0
P4—Female, 60–70 years	13	13	0
P5—Female, 50–60 years	13	13	0
P6—Male, 50–60 years	10	10	0

^a^ Modified test, change therefore not relevant.

#### Canadian Occupational Performance Measure

The patient scores in COPM after the rehabilitation program were overall higher at the end of the course compared with at the beginning of the course. The satisfaction of the performance of the chosen activities had increased significantly, with a score of ≥3.5 (34) for four of the six patients (Table 3).

**Table 3:** Test results of COPM.

COPM	* *	Pre	Post	Change
P1—Male, 40–5 years	Performance	3.8	6	2.2
	Satisfaction	1.6	4.8	3.2
P2—Male, 40–50 years	Performance	5.4	6.6	1.2
	Satisfaction	1.6	5.4	3.8
P3—Male, 70–80 years	Performance	3.6	7.4	3.8
	Satisfaction	2.4	7.2	4.8
P4—Female, 60–70 years	Performance	3.5	6	2.5
	Satisfaction	3	7.75	4.75
P5—Female, 50–60 years	Performance	3.6	7.2	3.6
	Satisfaction	1.8	7	5.2
P6—Male, 50–60 years	Performance	5.8	6	0.2
	Satisfaction	3.6	6	2.4

## Discussion

The novelty of this study is in the First Step approach and in the group of patients, who did not receive any form of rehabilitation or education in the course of their current treatment. The novelty is also in its strong focus on tailoring the intervention to best accommodate the patients’ individual needs. In the following we will initially discuss the outcome of incorporating the concept of First Step and thereafter discuss the adaptations that were made to the intervention. Ultimately, the strengths and limitations of the study are discussed.

In using the First Step approach the therapists were inspired to focus on the patients’ resources and ability to manage the challenges that come with the affliction of a chronic disease such as sleep apnea. The focus on SOC and what is experienced as meaningful by the individual has in other studies been linked to COPM and how it can be used in facilitating the process in which the patient comes to terms with life (35). In this study, this was reflected in the results of COPM. Every patient had a positive change on both performance and on how satisfied they were with the way they performed the chosen activities. The cause of this improvement could be found in the interventions’ focus on individual activity problems and ways to better these with actions that correlate with the patients’ everyday lives. This granted favorable conditions for the patients to experience a greater satisfaction with their own performance of daily activities.

The eye-opening experience that several patients reported could be a result of analyzing ADL in a structured way. The powerful recognition of how they have constructed their everyday life was both confronting and liberating to some and opened the possibility to make conscious changes to their way of living and thereby strengthen their self-efficacy.

The use of COPM and the MOHO-inspired approach also helped the multidisciplinary team get to know the patients in a more comprehensive way. This promoted the collaboration between therapist and patient in goal-setting, the motivational process, and when working with strengthening self-efficacy during the course (35). This again increased the possibility of the patient engaging in physical activity and physical training in a way that made sense to them. Future studies would benefit from a structured monitoring of self-efficacy from beginning to end, as strengthening self-efficacy may have a pivotal impact on an individuals’ ability to stay motivated and engaged in physical activity (36).

Even though the intervention was focused on individual activity problems, patients were encouraged to go for walks and were able to track their daily step count with an activity tracker. This approach led to a significant increase in the 6MWT scores for some of the patients, with no difference in the Sit-to-Stand test. The increased walking endurance may be a result of the supervised workout sessions twice a week, contributed by the external motivation from the activity tracker as well as the patients’ work with energy distribution tools.

The First Step concept allowed occupational and physiotherapy to merge into a very favorable mix. The therapeutic approach focused on the active engagement and involvement of the patients rather than just providing information and care. The active engagement was considered pivotal for the patients to take responsibility for their way of living and for maintaining good habits after the course. Another study by Garvey et al. has taken a similar yet different OT-driven approach that centers primarily on managing multimorbidity and the challenges this brings to everyday life (37). With our study's strong focus on also incorporating PT-based activities and exercises in a graduated way, it is the authors’ belief that the patients’ stood to gain not only on the ability to manage stressors but also objectively on physical functions. In future studies comprehensive tests to monitor effects on physical function would be necessary to fully explore how the First Step concept affects patients.

The patients were included in the planning and setting of the interventions. This study's explorative approach made it possible to evaluate two commonly used interventions; thereby creating broader experience-based findings. Patient experiences and opinions were used to help guide the best possible framework for a First Step–inspired sleep apnea program.

When evaluating the intervention, common topics among patients were the length and frequency of the sessions. Patients attending the rehabilitation program would prefer fewer sessions, but of a longer duration. This was to support social elements of the class and create better time for individualization in guidance of energy management and exercise. Patients attending the patient education preferred more sessions at the cost of a shorter duration.

A change in the numbers of sessions per week was made in part to accommodate patients who had barriers to participation due to transportation to and from the hospital. This change could potentially be avoided if transport was offered to patients experiencing logistical difficulties. This was not an option during this study.

When using the First Step concept as an approach to rehabilitate similar patient groups, findings suggest the patients prefer a course that includes multidisciplinary education and rehabilitation as well as more time set aside to accommodate individual needs.

The feedback indicates that an optimal intervention should be of 8–12 weeks with a weekly session of 1.5 h to ensure ample time to consider individual needs.

The intervention should include the following:

- A COPM interview pre and post intervention to establish meaningful goal-setting.
- Patient education in energy management, treatment of sleep apnea, pathology and guidance in use of CPAP from a team of multidisciplinary health professionals.
- Physical exercise during the weekly session as well as home-based exercises chosen to support the initial goal-setting for the individual.
- Homework related to energy management, with follow-ups during the course, to ensure experience with practical use of the tools.

The concept of First Step is built on experiences with different rehabilitation and education programs and is set to help each patient take the first step toward a better quality of life, despite reduced energy. Other relevant participants could be patients with one or several chronic diseases that lead to fatigue or with other personal barriers to being physically active.

The study is primarily evaluated through the experiences of the patients, although the supplementary quantitative measurements may give an indication toward a future research focus. In this study the First Step concept has helped patients with sleep apnea achieve increased walking endurance and a higher overall satisfaction with daily activities. Although the quantitative data is not statistically significant, it is the opinion of the authors that these results may indicate a potential positive effect in using of similar intervention to support patients in being increasingly active, potentially lose weight, and thereby experience decreased sleep apnea symptoms in general. Further investigation of this perspective could be beneficial in future studies.

The strength of the study lies in the use of the novel concept First Step, bringing a salutogenic approach to healthcare through patient education and empowerment, based on the individual's current situation. The concept aims to support meaningful changes in a patient's everyday life to give them the resources to be more physically active. In the intervention, actions were undertaken to meet the individual needs of the patients by having a meaningful goal-setting and working toward or incorporating exercise, e.g., by walking their dog in shifting intervals of high and low intensity. Based on the initial individualization the OT and PT made weekly schedules for every patient, helping them make time and conserve energy for both exercise as well as daily activities.

The study has limitations in being primarily qualitative in nature, thereby not being able to measure any effect of a specific intervention. The study aimed to mitigate the risk of desirability bias during interviews conducted at the hospital. To achieve this, patients did not meet the interviewer, who also dressed in plain clothing. Furthermore, the study's time frame and evaluation are on short-term changes. In the hopes of making a long-term change in patients’ daily living, this study's interventions would focus on incremental, but sustainable, changes in physical activity. A study from Karlsen et al. found that positive effects of a 12-week exercise intervention were lost at the 24-week follow-up most likely due to detraining (38). It is the authors’ opinion that this study would have benefitted from monitoring the effect on quality of life as well as a follow-up period to determine if the effects of the intervention were sustainable long term. This study would profit greatly from a consideration of a larger sample size focused on the effects of the intervention to combine both quantitative and qualitative findings in the evaluation of the First Step–inspired model.

A thorough intervention for the management of chronic fatigue is important, since fatigue can potentially contribute to the development of atherosclerosis, frailty, and sarcopenia. Fatigue can lead to a sedentary lifestyle and reduced physical activity due to a lack of energy. Sedentary behavior is a known risk factor for atherosclerosis as it can contribute to conditions such as obesity, high blood pressure, and diabetes (39). These conditions can accelerate the development of atherosclerosis, a disease where plaque builds up inside your arteries, leading to heart disease and stroke.

Furthermore, reduced physical activity can result in muscle wasting and weakness, key components of frailty (39). Fatigue can also lead to decreased social engagement and cognitive decline, both of which are associated with frailty (39).

With the loss of muscle mass due to inactivity, patients are at risk of developing sarcopenia, characterized by the loss of muscle mass and function. Sarcopenia can in itself lead to a decreased physical activity and a more sedentary lifestyle (40).

Fatigue, atherosclerosis, frailty, and sarcopenia are all conditions that can be a result of sedentary behavior as well as be contributors to further inactive lifestyles. This emphasizes the need to focus on how fatigue is best managed and physical activity facilitated in activities of everyday life. In this way the focus on fatigue management in combination with physical activity could potentially contribute to the prevention of multiple conditions.

Other studies have focused on the impact of an intervention on mood and cognitive disorders in patients with sleep apnea (41). In this study, the main focus has been on ADL and physical exercise, whereas a supplementary focus on mood and cognition would have broadened the perspective and would be a contribution to the assessment of the intervention.

This study focuses on fatigue management but does not take into account potential confounders such as malnutrition. Nutrition warrants an important role in the context of fatigue management. Recent studies have highlighted the established mechanism that underlies the connection between nutrition and fatigue (42). Certain nutrients have been implicated in various physiological processes that may influence the onset of numerous pathologies, including fatigue (42).

Moreover, the relationship between nutrition and malabsorption in atherosclerosis, stroke, and sarcopenia is crucial (43–45). Nutrients can affect the occurrence and development of atherosclerosis, which is the basic pathological process of many diseases, such as coronary atherosclerosis and stroke (43).

Therefore, while our study primarily focuses on occupational therapy and physiotherapy interventions for fatigue management, it is essential to acknowledge the potential influence of nutritional factors. Future interventions could potentially incorporate nutritional counseling or dietary modifications as part of a comprehensive approach to fatigue management. This could help address potential confounders such as malnutrition and provide a more holistic treatment strategy. Further research is needed to explore the integration of nutritional interventions in fatigue management protocols and their impact on patient outcomes.

The study uses this First Step–inspired model on patients with sleep apnea. It is the authors’ strong belief that similar intervention is likely to be just as effective on other patient groups experiencing fatigue in such a way that it limits their abilities to be physically active. Additional research on other patient groups is necessary to explore this further.

This study is conducted as part of outpatients’ treatment at a hospital. To accommodate patients’ wishes for a continued peer support group, implementation of a similar program should be anchored at local healthcare centers.

## Conclusion

In summary, our study demonstrates the usability of the First Step concept in constructing and implementing diverse interventions. By integrating occupational therapy and physiotherapy, this salutogenic approach addresses the unique challenges faced by individuals with sleep apnea. The tailored, multidisciplinary intervention prioritizes meaningful activities, energy distribution, and physical exercise, leading to improved satisfaction and performance. While positive outcomes are evident, further research is needed due to the lack of quantifiable data. Refining the First Step concept holds promise for enhancing rehabilitation programs not only for patients with sleep apnea but also for those with other chronic conditions.

## Data Availability

The original contributions presented in the study are included in the article/Supplementary Material, further inquiries can be directed to the corresponding author.
